# On the Thermomechanical Behavior of 3D-Printed Specimens of Shape Memory R-PETG

**DOI:** 10.3390/polym15102378

**Published:** 2023-05-19

**Authors:** Ștefan-Dumitru Sava, Nicoleta-Monica Lohan, Bogdan Pricop, Mihai Popa, Nicanor Cimpoeșu, Radu-Ioachim Comăneci, Leandru-Gheorghe Bujoreanu

**Affiliations:** Faculty of Materials Science, “Gheorghe Asachi” Technical University of Iași, Blvd. Dimitrie Mangeron 71A, 700050 Iasi, Romania; stefan-dumitru.sava@student.tuiasi.ro (Ș.-D.S.); nicoleta-monica.lohan@academic.tuiasi.ro (N.-M.L.); bogdan.pricop@academic.tuiasi.ro (B.P.); mihai.popa@academic.tuiasi.ro (M.P.); nicanor.cimpoesu@academic.tuiasi.ro (N.C.); radu-ioachim.comaneci@academic.tuiasi.ro (R.-I.C.)

**Keywords:** recycled PETG, 3D printing, shape memory effect, storage modulus, work generation, fractographs

## Abstract

From commercial pellets of recycled polyethylene terephthalate glycol (R-PETG), 1.75 mm diameter filaments for 3D printing were produced. By varying the filament’s deposition direction between 10° and 40° to the transversal axis, parallelepiped specimens were fabricated by additive manufacturing. When bent at room temperature (RT), both the filaments and the 3D-printed specimens recovered their shape during heating, either without any constraint or while lifting a load over a certain distance. In this way, free-recovery and work-generating shape memory effects (SMEs) were developed. The former could be repeated without any visible fatigue marks for as much as 20 heating (to 90 °C)-RT cooling–bending cycles, while the latter enabled the lifting of loads over 50 times heavier than the active specimens. Tensile static failure tests revealed the superiority of the specimens printed at larger angles over those printed at 10°, since the specimens printed at 40° had tensile failure stresses and strains over 35 MPa and 8.5%, respectively. Scanning electron microscopy (SEM) fractographs displayed the structure of the successively deposited layers and a shredding tendency enhanced by the increase in the deposition angle. Differential scanning calorimetry (DSC) analysis enabled the identification of the glass transition between 67.5 and 77.3 °C, which might explain the occurrence of SMEs in both the filament and 3D-printed specimens. Dynamic mechanical analysis (DMA) emphasized a local increase in storage modulus of 0.87–1.66 GPa that occurred during heating, which might explain the development of work-generating SME in both filament and 3D-printed specimens. These properties recommend 3D-printed parts made of R-PETG as active elements in low-price lightweight actuators operating between RT and 63 °C.

## 1. Introduction

Glycol-modified polyethylene terephthalate was developed with the aim of increasing the durability and strength of polyethylene terephthalate (PET), thus contributing to the improvement of both impact and high-temperature resistance [1]. These attributes have recommended polyethylene terephthalate glycol (PETG) as a raw material for textiles, beverage bottles, packaging materials, and 3D printing filaments [2].

Owing to the higher glass transition interval, lower melting temperature, and viscosity of PETG compared to PET [3], better results were obtained with the use of the former as a raw material to make filaments for 3D printing [4,5]. Consequently, various complex parts were 3D printed from PETG using different additive manufacturing techniques [6,7,8].

Considering that both PET and PETG experience a glass transition [9], which can cause the occurrence of shape memory effects (SMEs) characterized by the recovery of a permanent shape by heating the material with a deformed temporary shape [10], it is expected that both polymers undergo thermal memory phenomena [11]. The occurrence of SMEs in a PETG filament for 3D printing is illustrated in Video S1 from the Supplemental Material. Consequently, shape variation with time was regarded as the fourth dimension, which originated 4D printing [12] as an additive manufacturing field that uses time-responsive programmable materials [13] and has the potential to develop a wide range of adaptable shape applications [14]. For example, 3D printing has successfully been used for repairing lightweight automotive components manufactured from carbon fiber-reinforced polymers [15], for the production of water nanofiltration membranes [16], and for in vitro diagnosis applications [17].

In the particular case of PETG, owing to its special features, novel applications with highly controllable self-coiling and tensile shape memory behavior have been elaborated [18], with the precise tuning of the effect of programming temperature [19].

Aiming to further increase the mechanical properties of PETG-printed specimens, various experimental approaches have been used, including aging-induced relaxation [20], accurate control of overlap ratio parameters [21], layer spatial orientation [22], and the use of complex architectures, such as honeycomb structures [23]. However, increasing mechanical resistance at room temperature (RT) in the amorphous state might decrease the magnitude of SME due to difficulties in inducing a deformed temporary shape that is different from the permanent one [24].

One of the present major concerns is the necessity to recycle the huge amount of plastic waste accumulated in nature, mostly in the form of PET and PETG bottles [25]. To the best of the present authors’ knowledge, there are no reports on the study of SME in recycled PETG. Taking into account the huge recycling effort of PETG waste [26] and the previous results reported by some of the present authors on the shape memory properties of recycled PET (R-PET) [27], this article aims to investigate the performance of 3D-printed parts from filaments fabricated from this material.

## 2. Materials and Methods

**Figure 1:**
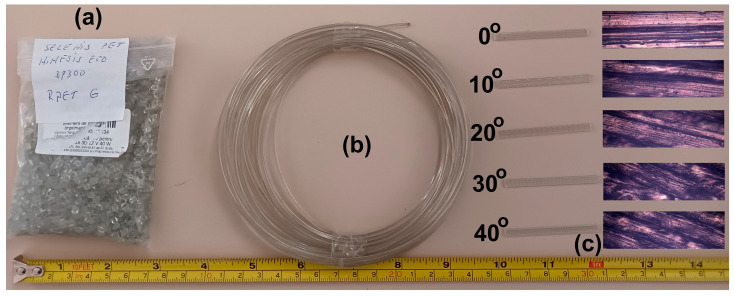
R-PETG raw materials and specimens: (**a**) R-PETG grains; (**b**) filament for 3D printing; (**c**) 3D-printed specimens, at different deposition angles to the transversal direction and OM details.

R-PETG grains were purchased from Selenis Company (Portalegre, Portugal). The grains were processed by heating, extrusion into filament, deposition, and cooling. The entire technology, including the experimental line for 3D filament production, was previously described in detail [27]. Table S1 in the Supplementary Material provides the printer’s specifications. Twenty-five 1 mm × 4 mm × 50 mm parallelepipedal specimens were printed; every five of them had different angles between the specimen’s transversal axis and the filament’s deposition direction (0°, 10°, 20°, 30°, or 40°), as illustrated in Figure S1 and Table S1 in the Supplementary Material. The images of the R-PETG raw material, filament, and printed specimens with optical microscopy (OM) details are shown in Figure 1.

In order to investigate the occurrence of SME, its evolution with the variation of the number of cycles, and the presence/absence of the applied load, several filament fragments and 3D-printed specimens were subjected to monitored heating–cooling cycles [27]. For this purpose, the specimens were fastened at one end, bent to 90° at room temperature (RT), heated with a hot air gun to 90 °C, and cooled in air to RT, either without any constraint or with a load fastened at their free end. Thus, free-recovery or work-generating SMEs were developed [28]. For this purpose, a special fastening device for the filaments was prepared that was capable of being loaded with different weights. Several 1.75 mm diameter filament fragments were cut into 50 mm lengths. Their weight was 0.1724 g. The fastening device was loaded with two different weights in such a way that the total load masses were 5.723 g and 9.981 g. The evolutions of the vertical position of the specimen’s free end during heating were monitored by cinematographic analysis and examined frame by frame [29]. The measuring precisions were ±1 mm for vertical displacement and ±2 °C for temperature.

**Figure 2:**
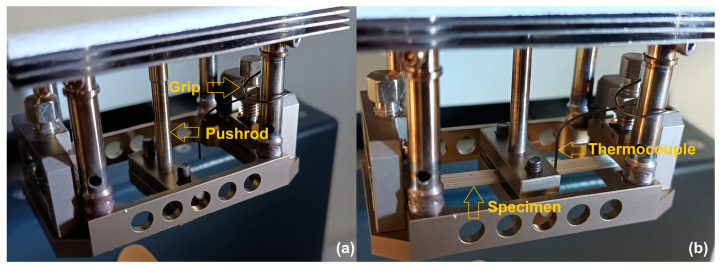
Images of the dual-cantilever specimen holder of the DMA device: (**a**) without specimen; (**b**) with R-PETG specimen fastened in the pushrod’s grips and lateral grips.

Thermal analysis was performed using differential scanning calorimetry (DSC) and dynamic mechanical analysis (DMA) with a NETZSCH DSC 200 F3 Maia device (Netzsch, Selb, Germany) calibrated with Bi, In, Sn, Zn, and Hg standards and a NETZSCH DMA 242 Artemis device (Netzsch, Selb, Germany) equipped with a dual-cantilever specimen holder, respectively, using the previously detailed procedure [27]. The accuracy of the DSC device is given by the following parameters: digital resolution: 0.2 µW/digit; signal noise: 0.7 µW RMS at 130 °C (equivalent to 3.6 µW peak-to-peak); signal time constant: >2.5 s (indium melting peak); reproducibility onset temperature: +/−0.1 K (indium melting peak); and reproducibility of peak areas: +/−1% (indium melting peak). The technical data of the DMA device comprise the following parameters: data acquisition resolution: ±2,000,000 digits, rate 20 s^−1^, and sensitivity ±2.5… ±10 V; sample temperature resolution: 0.01 °C; and accuracy > 0.5 °C. Figure 2 shows images of the dual-cantilever specimen holder and the fastening details of the specimen.

The 3D-printed R-PETG specimen was fastened both in the lateral grips and in the pushrod’s grip by means of a torque wrench.

The final set of experiments was meant to reveal the behavior of the 3D-printed specimens during static tensile failure tests and the accompanying structural changes as a function of the angle between the specimen’s transversal axis and filament deposition direction. For this purpose, five specimens (type ISO 527-2/1A/50) with the configuration shown in Figure S1 in the Supplementary Material were 3D printed with each of the five above-mentioned angles between the specimen’s transversal axis and filament deposition direction (between 0° and 40°). The five specimens from each of the above-mentioned batches were subjected to tensile tests performed at RT with an INSTRON 3382 tensile machine (Norwood, MA, USA) and a cross-head speed of 1 mm/min. The machine has the following features: 100 kN (22,500 lbf) capacity; maximum speed 508 mm/min (20 in/min); 1430 mm (56.3 in) vertical test space; and ±0.5% of reading down to 1/200 of load cell capacity and ±1% of reading from 1/200 to 1/500 of the load cell capacity. The tensile strain was measured with an INSTRON 2620 clip-on extensometer with a gauge length of 25 mm and a linearity of 0.15/full scale. On the failure cross-sections, a 10 nm-thick gold layer was deposited with a LUXOR Au/Pt Coater (APTCO, Berlin, Germany), and scanning electron microscopy (SEM) fractographs were recorded using a VEGA II LSH TESCAN device (TESCAN, Brno—Kohoutovice, Czech Republic).

## 3. Results and Discussion

### 3.1. Shape Memory Effect

**Figure 3:**
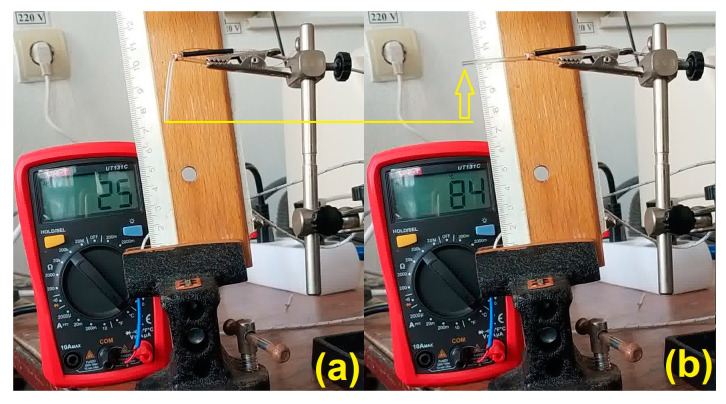
The occurrence of free-recovery SME of the R-PETG filament: (**a**) RT bent to a temporary shape; (**b**) permanent shape recovery during heating and illustration of free-recovery SME.

Two representative images of the cold (temporary) and hot (permanent) shapes of the filament during free-recovery SME development are illustrated in Figure 3.

**Figure 4:**
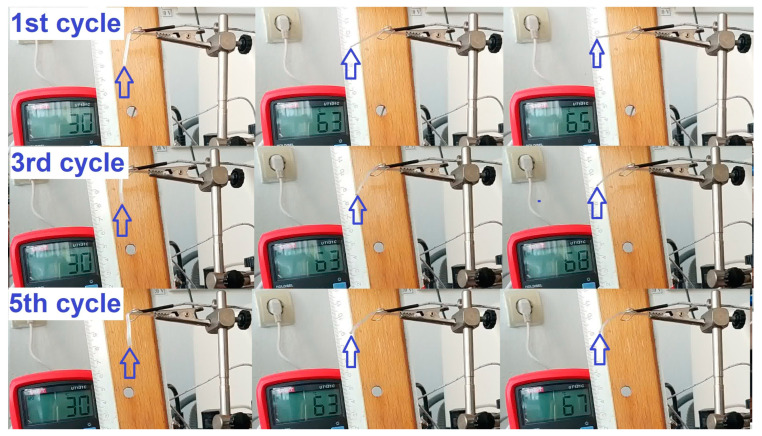
Recorded images illustrating the specimen’s free-end displacement during three heating–cooling–straightening–bending cycles applied to R-PETG filaments (see text for details).

It is noticeable that when the temperature increased by 59 °C, from 25 to 84 °C, the R-PETG filament fragment developed free-recovery SME, characterized by a vertical displacement of 28.7 mm. However, when heating was continued, even by a few degrees, the filament totally lost its stiffness and re-became bent. After the heating stopped, the filament had to be straightened and kept in this position until its temperature dropped to 40 °C (by simple air cooling) when it regained its stiffness. Then, it could be bent again, and the procedure could be resumed. This behavior was not noted with R-PET, which was unable to develop SME with the same recovery degree in the second cooling—bending–heating cycle [27]. Aiming to observe if R-PETG can develop reproducible free-recovery SME, five cycles were applied, and the typical images of three cycles are summarized in Figure 4.

**Figure 5:**
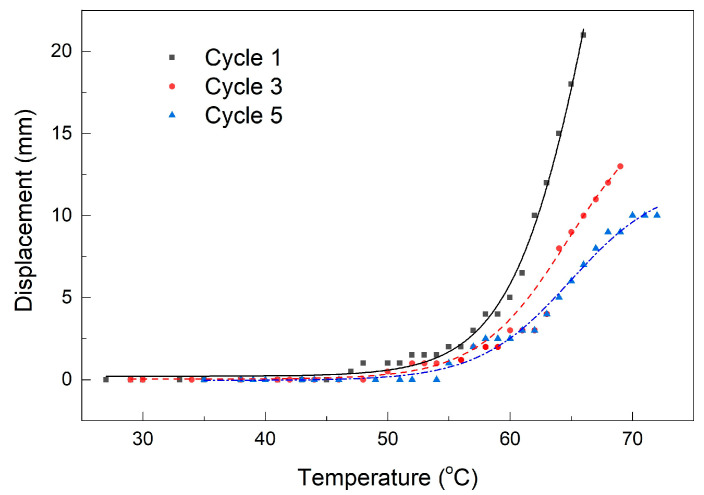
Variations of free end’s displacement vs. temperature during three free-recovery SME cycles, according to Figure 4.

The images show the filament’s positions at RT, at 63 °C, and at temperatures where the maximum strokes were developed. By cinematographic analysis, the free end’s positions and the filament’s temperatures were determined, frame by frame, and the displacement vs. temperature variations were plotted for the first, third, and fifth cycles, as illustrated in Figure 5.

The symbols correspond to experimental values, and the solid lines to the Boltzmann fitting functions that were also used in the study of R-PET with the form [27]:

(1) $$y=A_{2}+(A_{1}-A_{2})/(1+e^{\frac{x-x_{0}}{\mathrm{dx}}})$$

where y = displacement (mm) and x = temperature (°C).

Table 1 lists the values of the parameters of the Boltzmann function, together with their errors for the three cycles illustrated in Figure 5.

In Table 1, the derived parameters represent: abs(A1 − A2)—*span* and half maximal effective concentration—*EC50* = exp(x_0_). *Red. Chi-Sqr*—reduced chi-squared value and *Adj. R-Square*—a modified version of R-Square is used to evaluate the fitted function’s suitability. It is noticeable that the largest errors are present only in Cycle 1 and only in the case of the parameters final value, A_2_ and *span*. These results confirm that the variation of the free end’s displacement as a function of temperature due to free-recovery SME development both at R-PET and R-PETG can be fairly modeled by means of a Boltzmann function.

**Figure 6:**
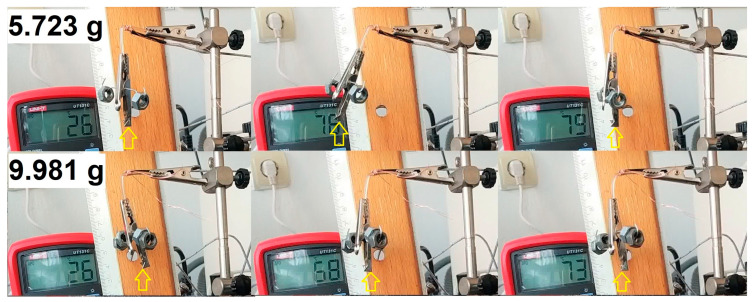
Recorded images illustrating the development of work-generating SME during the heating of R-PETG filaments with two different applied loads.

The next series of experiments was meant to emphasize the capacity of the R-PETG samples to develop work-generating SME. Consequently, two filament fragments were bent at RT, and two different loads were fastened at their free ends by means of the special fastening device for filaments; then, they were heated, and the entire development was filmed. The representative images are depicted in Figure 6.

Both applied loads were over 33 and 57 times heavier than the R-PETG filament fragment. Consequently, the vertical lifts were much lower than in the case of free-recovery SME development.

**Figure 7:**
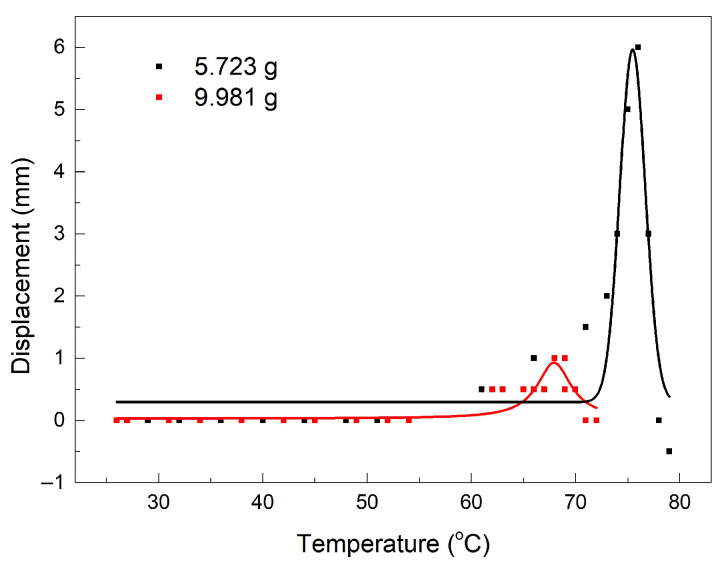
Variations in the free end’s displacement vs. temperature during work-generating SME, developed by R-PETG filament, according to Figure 6.

The variations in the free end’s displacement as a function of temperature, determined by cinematographic analysis, are illustrated in Figure 7.

In this case, the softening caused by the heating procedure, beyond the point of maximum stroke development, caused a sharp displacement decrease (the load was lowered even below the initial point), and for this reason, the Boltzmann fitting function was no longer applicable. The only candidate for the fitting of the experimental values of the free end’s displacement vs. temperature was a Lorentz function, which has the form:

(2) $$y=y_{0}+\left(\frac{2A}{\pi }\right)\left[\frac{w}{4{\left(x-\mathrm{xc}\right)}^{2}+w^{2}}\right]$$

The parameters from Equation (2), together with their corresponding standard errors, are listed in Table 2.

The very large standard errors, noticeable at parameters xc, A, and H, for the applied load of 5.723 g demonstrate that the Lorentz function was not suitable for fitting these data. In the case of the 9.981 g applied load, all standard errors were below 1.3%.

### 3.2. Thermal Analysis

**Figure 8:**
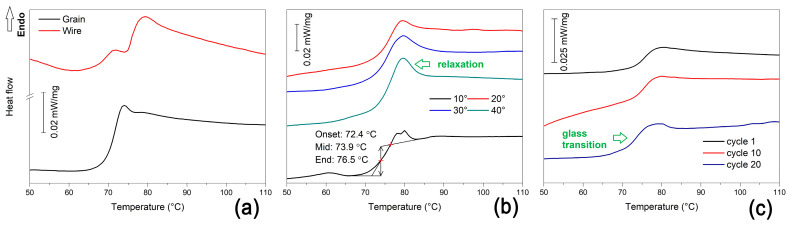
Typical DSC charts recorded during the heating of fragments cut from various R-PETG samples: (**a**) grains/pellets and filament/wire; (**b**) 3D-printed specimens at various deposition angles to the transversal direction; (**c**) specimens subjected to different numbers of free-recovery SME cycles.

Figure 8 summarizes the representative DSC charts obtained during the heating of the R-PETG grains or fragments cut from filaments and 3D-printed specimens. Each chart reveals at least one endothermic step that is associated with a glass transition, while the filament reveals two such transformations.

These glass transitions represent the microstructural mechanism of SME during which the amorphous regions of R-PETG are transformed into crystalline phases [10]. The presence of small endothermic peaks at the end of the glass transitions can be noticed in most of the DSC charts. They have been associated with structural relaxation phenomena [30]. Both glass transition and structural relaxation are pointed out by arrows.

It is noticeable that the increase in both the deposition angles and the number of SME cycles enhanced structural relaxation. The results of the glass transition evaluation are listed in Table 3.

As noticed in Figure 5, the temperature of maximum stroke developed by free-recovery SME had the tendency to increase with the number of applied cycles. This could be an effect of the straightening imposed on each filament sample during air cooling between 90 °C and 40 °C, after which they recovered their stiffness and could be bent again at RT. A deeper study of the behavior of 3D-printed parts of R-PETG will be the subject of another article.

The temperature values from Table 3 show that the glass transition thermal range of R-PETG was not strongly influenced by the printing angle and the number of free-recovery SME cycles. When comparing the above values with those found for R-PET, it is obvious that R-PETG (i) experienced glass transition in a lower thermal range and (ii) absorbed more energy during glass transition.

The next series of thermal analysis experiments was performed using DMA, either during heating under constant bending frequency or during isothermal maintaining at RT, while applying different bending frequencies.

**Figure 9:**
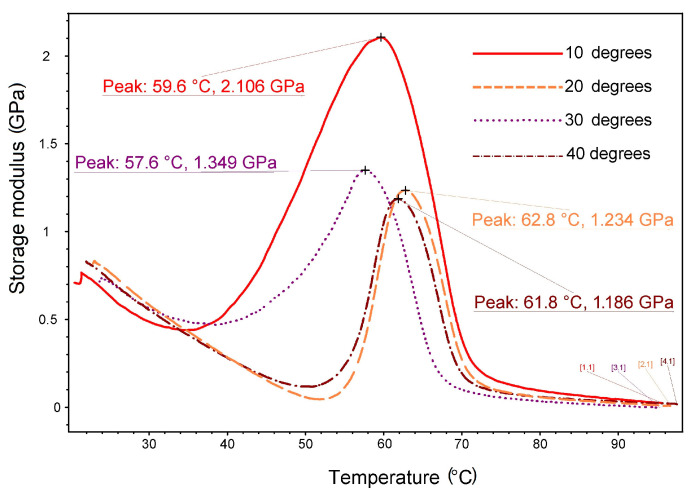
Storage modulus variations vs. temperature during the heating of R-PETG 3D-printed specimens at various deposition angles to the transversal direction.

The typical DMA diagrams recorded during the heating of 3D-printed specimens subjected to a dynamic bending frequency of 1 Hz are shown in Figure 9.

The sharp decrease in storage modulus that is typically associated with glass transition [9] was preceded by marked stiffening when the storage modulus increase reached between 0.87 GPa for the specimen printed at 30° and 1.66 GPa for the specimen printed at 10°. These large values, almost double those observed for R-PET [27], could be a logical explanation for the capacity of R-PETG to develop work-generating SME.

The values of the storage modulus maxima shown in Figure 9 ranged between 1.18 and 2.1 GPa, which is in good agreement with the literature [31,32]. Their corresponding temperatures located between 57.6 and 62.8 °C were lower than the onset of the glass transition from Table 3. This is due to the lower heating rate of DMA (3 °C/min) than that of DSC (10 °C/min), as it is well known that critical temperatures increase with increasing the heating rate [33].

**Figure 10:**
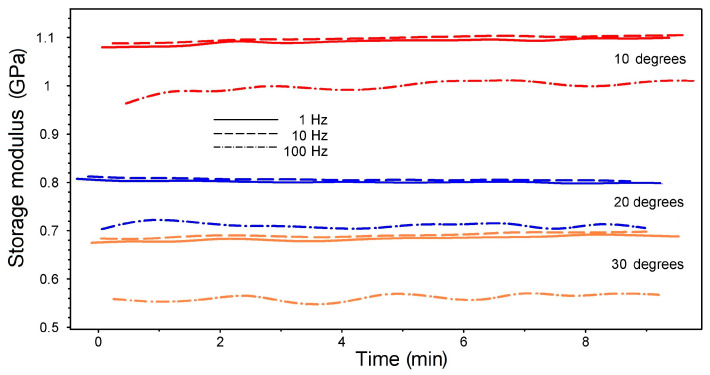
Storage modulus variations vs. time during DMA-isothermal strain sweeps at RT of R-PETG 3D-printed specimens at various deposition angles to the transversal direction.

The DMA tests with isothermal maintaining were performed at RT using three dynamic bending frequencies: 1, 10, and 100 Hz. These dynamic isothermal tests were designed to monitor the frequency’s influence on the material’s behavior [34]. The variations in storage modulus over time for the three 3D-printed specimens with different deposition angles (10°, 20°, and 30°) are presented in Figure 10.

The diagram shows that the storage modulus remained almost constant for 9 min, during which over 50,000 dynamic bending cycles were applied at a frequency of 100 Hz. Similar behavior was observed for the 3D-printed specimens from R-PET [27].

It is noticeable that increasing the frequency 10 times did not have a marked effect, but increasing it 100 times caused storage modulus variations as high as 0.13 GPa. The highest storage modulus values were obtained for the lowest deposition angles to the transversal direction. This effect could be explained by the augmentation, with the increase of the angle to the transversal direction, of the length of the fibers with fixed ends that were bent by the pushrod. Increasing the length of a solid with fixed ends subjected to bending under constant force would normally cause an increase in bending deflection due to the proportionality between deflection and length [35].

It follows that, from the point of view of bending, increasing the angle between the deposited filament layers and the transversal direction caused a softening accompanied by a modulus decrease.

### 3.3. Tensile Behavior

**Figure 11:**
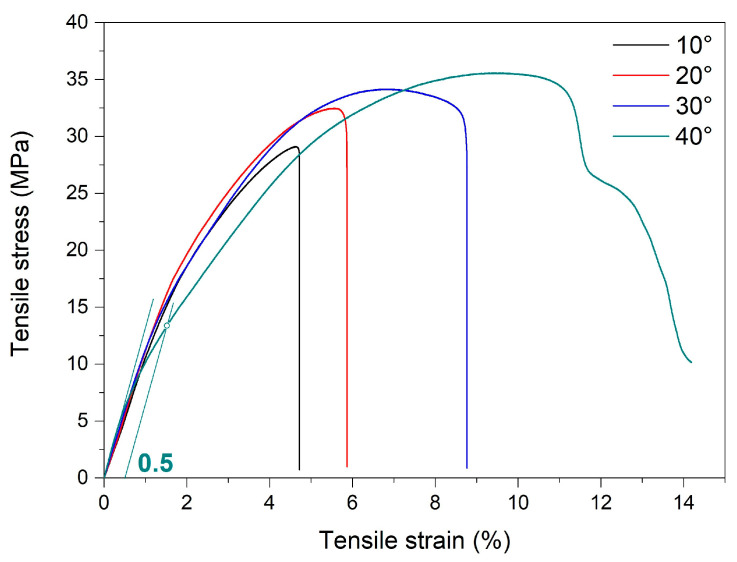
Representative tensile failure curves at RT of R-PETG 3D-printed specimens at various deposition angles to the transversal direction.

The static tensile failure curves of the 3D-printed specimens are illustrated in Figure 11.

The figure displays the offset yield point of 0.5%, according to ISO 527-2 [36]. The calculated values of the main mechanical parameters are summarized in Table 4.

The average tensile failure stresses were located between approximately 29 and 35 MPa. These values were higher than those reported for recycled polymer materials from post-process wastes [31] but similar to those obtained with specimens that were 3D printed from filaments fabricated with a twin screw extruder [32] or injection mold from scraped foils [37].

With increasing the angle between fiber deposition and transversal direction, both failure stress and ultimate strain increased because both the number of adjacent layers that were simultaneously deformed [38] and their bonding area increased [39]. In other words, by increasing the angle between fiber deposition and transversal direction, the failure mode shifts from interlayer failure or delamination to rupture in the grid, as will be shown later.

**Figure 12:**
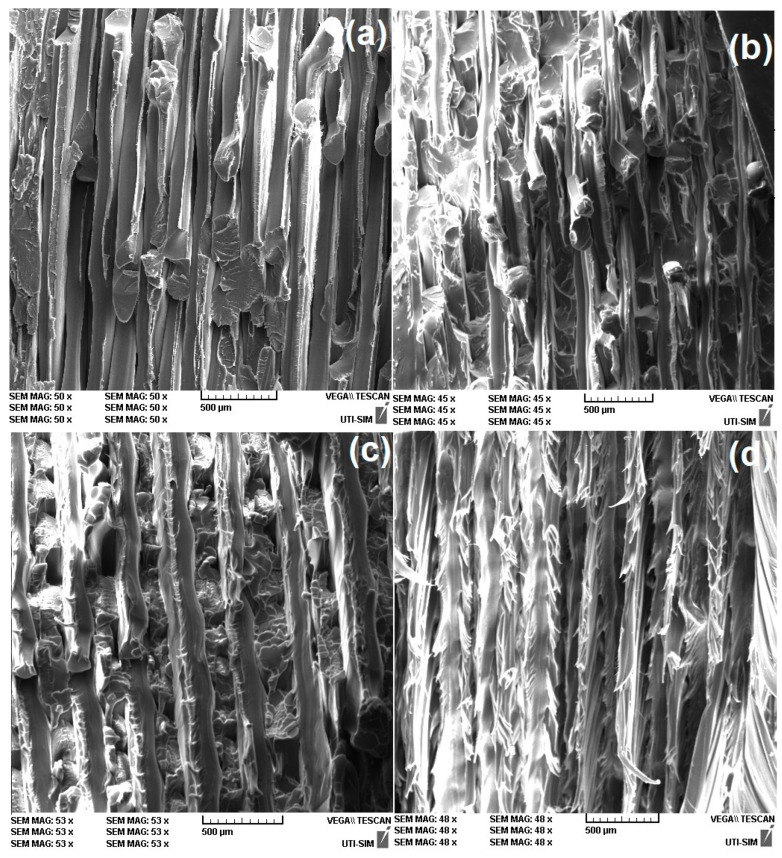
SEM fractographs of the cross-sections of R-PETG 3D-printed specimens at various deposition angles to the transversal direction that failed, according to Figure 11: (**a**) 10°; (**b**) 20°; (**c**) 30°; and (**d**) 40°.

The maximum value of the ultimate strain was only approximately 14% for the specimen printed at 40° to the transversal direction, which is rather different from the demonstrated values by the high deformation capacity of PETG [40]. In order to explain this discrepancy, a fractographic study was performed on the cross-sections of the tensile broken specimens. The representative fractographs are shown in Figure 12.

It is known that during tensile failure, fibers are pulled out from the polymeric matrix [41]. With increasing the angle between filament deposition and transversal direction, fewer fibers were pulled out, while more and more became torn and shredded. At larger angles, more fibers were individually broken and shredded instead of being delaminated.

**Figure 13:**
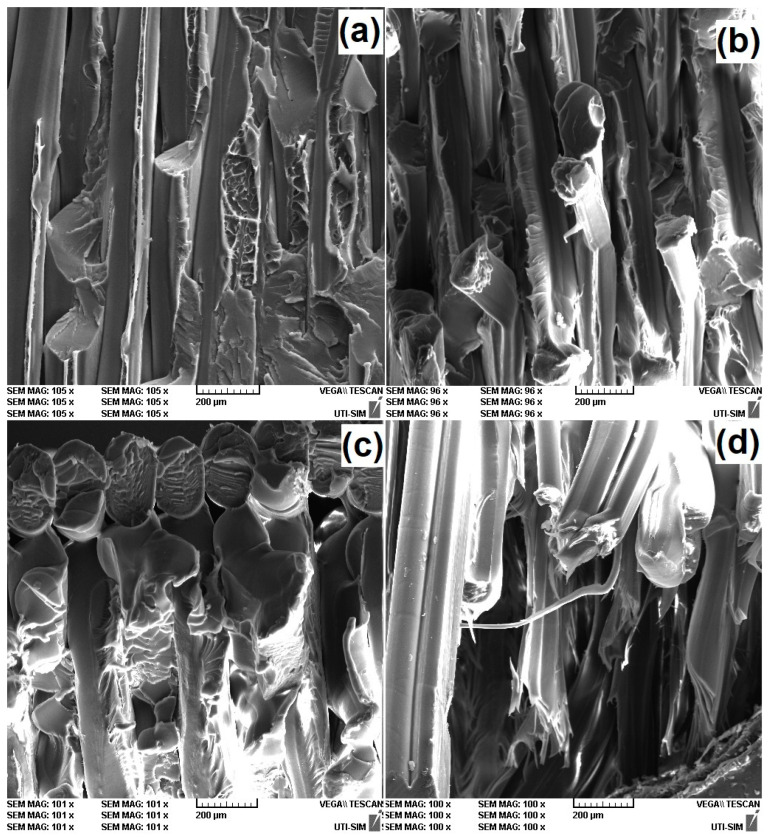
Higher magnification SEM fractographs of the cross-sections from Figure 12: (**a**) 10°; (**b**) 20°; (**c**) 30°; and (**d**) 40°.

With increasing the deposition angle, both tensile failure stress and ultimate strain increased. These increases can be explained by the augmentation of both the number of simultaneously deformed adjacent layers and their bonding area. However, closer consideration is necessary to explain why the ultimate strain was rather limited and did not exceed 9%. For this purpose, higher magnification fractographs were recoded, as illustrated in Figure 13.

It is obvious that with increasing the deposition angle, more and more fibers are individually broken and shredded instead of successive layers being delaminated. In other words, it appears that the layers were unable to maintain their cohesion over larger strains when their bonding area increased [41].

## 4. Summary and Conclusions

The following activities were carried out: (i) producing continuous filaments from pellets of recycled polyethylene terephthalate glycol (R-PETG); (ii) testing the filaments’ capacity to develop both free-recovery and work-generating SME; (iii) analyzing the thermal behavior during DSC heating of pellets, filaments, and 3-D printed specimens with different deposition angles to the transversal direction; (iv) subjecting the 3-D printed specimens to DMA, both during single frequency heating and multi-frequency isothermal experiments; (v) performing RT tensile static failure tests; and (vi) analyzing fractographic 3-D printed specimens.

From each of the main types of experiments, the following conclusions were drawn while comparing the results from the present study to those previously obtained with R-PET:

### 4.1. The Capacity to Develop SME

- Both free-recovery and work-generating SME were emphasized in the case of filaments produced from R-PETG pellets and in the case of 3D-printed parts obtained from these filaments.
- The filaments experienced free-recovery SME for up to 20 consecutive cycles, during which the specimens were bent at RT, heated, and straightened during cooling after they lost their stiffness during heating.
- The variations of the specimens’ free-end displacement with temperature were fitted with Boltzmann type functions, which were applicable both for R-PET and R-PETG, with standard errors below 1.2% after the first cycle.
- Filament samples weighing 0.1724 g were able to lift a 9.981 g load over a 1 mm distance.

### 4.2. Thermodynamic and Dynamic Mechanical Behavior during Heating

- R-PETG experienced glass transition at lower temperatures and absorbed more energy compared to R-PET.
- By increasing both the filament deposition angle to the transversal direction and the number of free-recovery SME cycles (up to 20), structural relaxation was enhanced.
- DMA measurements during heating performed with dual-cantilever dynamic bending emphasized storage modulus increases by up to 1.66 GPa, which might explain the capability of R-PETG to develop work-generating SME.
- Increasing the angle between the specimen’s transversal direction and layer deposition direction from 10° to 30° caused the storage modulus to decrease during RT DMA isothermal testing under constant force, which could be ascribed to the increase in the bending length of the fibers with fixed ends.

### 4.3. Static Tensile Failure

- Static tensile failure tests emphasized the increase in ultimate stress and strain when increasing the angle between fiber deposition and transversal direction, which could be caused by the augmentation of both the number of simultaneously deformed adjacent layers and their reciprocal bonding area.
- By means of SEM fractographs, with increasing the deposition angle, the failure mode shifted from interlayer failure or delamination to rupture in the grid, which might be the cause for maintaining ultimate tensile strains below 9%.
- All these results recommend 3D-printed parts of R-PETG for use as active elements in low-price lightweight actuators operating between RT and 63 °C.
- For a better understanding of R-PETG behavior during cooling, thermal analysis, tensile tests, and microstructural experiments are under development. Their results will be published in a subsequent article.

## Figures and Tables

**Table 1:** Values of the parameters of the Boltzmann function according to the fitting from Figure 5.

Parameter	Cycle 1		Cycle 3		Cycle 5	
	Value	Standard Err.	Value	Standard Err.	Value	Standard Err.
A_1_	0.21414	0.13531	0.03697	0.08628	−0.0425	0.14492
A_2_	56.58798	16.77323	16.98465	1.07996	12.1211	0.80303
x_0_	67.81872	1.77162	64.70565	0.53542	64.92739	0.61614
dx	3.55698	0.31073	3.65541	0.26207	3.7548	0.38831
span	56.37385	16.8312	16.94769	1.11499	12.16361	0.86625
EC_50_	2.84 × 10^29^	5.03 × 10^29^	1.26 × 10^28^	6.76 × 10^27^	1.58 × 10^28^	9.71 × 10^27^
Red. Chi-Sqr	0.19781		0.06259		0.16685	
Adj. R-Square	0.99422		0.99723		0.98798	

**Table 2:** Values of the parameters of the Lorentz function according to the fitting from Figure 7 for the two applied loads.

Parameter	5.723 g-Load		9.981 g-Load	
	Value	Standard Err.	Value	Standard Err.
y_0_	0.21414	0.30497	0.01737	0.05093
xc	56.58798	75.49819	67.93821	0.28424
W	67.81872	1.75038	3.97621	0.97137
A	3.55698	18.90585	5.70524	1.28991
H	56.37385	6.87614	0.91345	0.11626
Red. Chi-Sqr	0.6168		0.03166	
Adj. R-Square	0.76182		0.75166	

**Table 3:** Evaluation results of the thermograms from Figure 8.

Specimen	Onset, °C	Mid, °C	Inflection, °C	End, °C	ΔC_p_, J/(g∙°C)
grain	69.8	71.6	72.0	73.1	0.192
filament	74.9	76.4	75.6	77.6	0.141
printed at 10°	72.4	73.9	76.6	76.5	0.134
printed at 20°	73.5	76.2	76.4	78.3	0.157
printed at 30°	73.2	76.0	76.0	77.8	0.157
printed at 40°	73.6	75.6	77.3	77.4	0.161
1st cycle	73.2	76.0	76.0	78.3	0.133
2nd cycle	73.3	76.2	75.3	78.2	0.136
3rd cycle	68.8	72.8	72.3	75.8	0.235
4th cycle	67.5	70.2	70.4	73.3	0.091
5th cycle	69.3	72.1	73.0	74.7	0.186
6th cycle	71.1	73.8	74.0	75.7	0.222
7th cycle	70.0	73.1	74.0	75.5	0.217
8th cycle	69.0	72.6	72.6	75.6	0.195
9th cycle	72.3	74.7	74.8	76.5	0.243
10th cycle	68.9	72.6	71.3	75.0	0.182
11th cycle	68.2	71.9	72.0	75.1	0.199
20th cycle	70.8	74.7	73.6	77.1	0.212

**Table 4:** Mechanical parameters of the tensile failure curves from Figure 11.

Specimen Batch	Modulus	Tensile Stress at 0.5% Yield Offset	Tensile Strain at 0.5% Yield Offset	Tensile Failure Stress	Tensile Strain at Tensile Strength
	(MPa)	(MPa)	(%)	(MPa)	(%)
10°	1014.28 ± 35.34	22.48 ± 1.61	3 ± 0.1	29.09 ± 1.8	4.64 ± 0.32
20°	1073.97 ± 41.12	23.67 ± 1.74	3 ± 0.1	32.41 ± 1.85	5.66 ± 0.36
30°	1163.18 ± 40.36	19.71 ± 1.47	2 ± 0.1	32.76 ± 2.01	8.42 ± 0.42
40°	1337.47 ± 46.78	13.08 ± 1.92	1 ± 0.1	35.39 ± 2.11	8.76 ± 0.39

## Data Availability

Not applicable.

## Supplementary Materials

The following supporting information can be downloaded at https://www.mdpi.com/article/10.3390/polym15102378/s1: Figure S1: 3D-printed specimen geometry and deposition angle; Table S1: Summary of the values of printing parameters and printer’s specifications; and Video S1: Free-recovery SME of a fragment from an R-PETG filament.
