# Supplementary material for: On the Thermomechanical Behavior of 3D-Printed Specimens of Shape Memory R-PETG
Source: Polymers (Basel). 2023 May 19;15(10):2378. doi: 10.3390/polym15102378 (PMC10223659; doi:10.3390/polym15102378)
Supplement: Supplementary file 1 [file polymers-15-02378-s001.zip › polymers-2384555-supplementary - resub.pdf]

polymers

MDPI

Supplementary Materials

On the Thermomechanical Behavior of 3D-Printed Specimens of Shape Memory R-PETG

Ștefan-Dumitru Sava, Nicoleta-Monica Lohan, Bogdan Pricop, Mihai Popa, Nicanor Cimpoeșu, Radu-Ioachim Comăneci and Leandru-Gheorghe Bujoreanu *

Faculty of Materials Science, "Gheorghe Asachi" Technical University of Iași, Blvd. Dimitrie Mangeron 71A, 700050 Iasi, Romania; stefan-dumitru.sava@student.tuiasi.ro (Ș.-D.S.); nicoleta-monica.lohan@academic.tuiasi.ro (N.-M.L.); bogdan.pricop@academic.tuiasi.ro (B.P.); mihai.popa@academic.tuiasi.ro (M.P.); nicanor.cimpoesu@academic.tuiasi.ro (N.C.); radu-ioachim.comaneci@academic.tuiasi.ro (R.-I.C.)

* Correspondence: leandru-gheorghe.bujoreanu@academic.tuiasi.ro; Tel.: +40-727-486-406

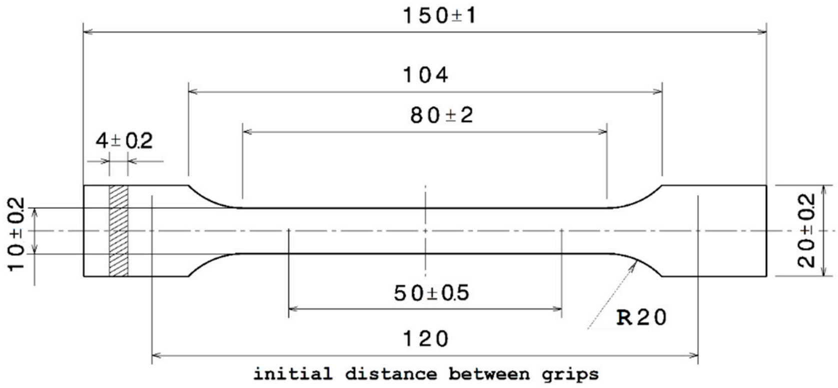

Specimen geometry according to EN ISO 527-2

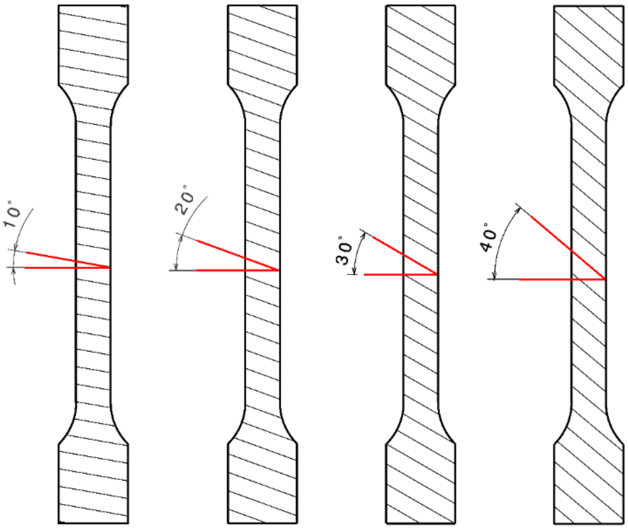

Illustration of the printing angle between transversal direction and filament deposition direction

Figure S1. 3D printed specimen geometry and deposition angle.

Table S1. Summary of the values of the printing parameters and printer's specifications.

	**Build Surface**	**Extruder temperature**	**Bed tempera-ture**	**Nozzle height**	**Printing speed**	**Travel speed**	**Cooling**
Printing parameters	PEI (Polyetherimide)	205-220 °C	60-70 °C	0.1-0.2mm	20-80mm/s	120mm/s	moderate
Printer's specifications	Style	Frame	Build volume, mm	Extruder configuration	Extruder	Accuracy	Speed
	CoreXY Cartesian	Aluminum extrusions with 3D printed parts	210 x 130 x 140	Direct	Bondtech BMG extruder (3:1 gear ratio)	0.2-0.4mm	20-130mm/s
	Bed leveling	Stepper motors	Voltage	Mainboard	Bed surface	Firmware	Computer
	manual leveling	NEMA 17	24 V (with Meanwell PSU)	SKR V1.4 with TMC2209 stepper motor drivers	PEI sheet	Klipper	Raspberry Pi 4 (to work with Klipper firmware)
